# Identification of MNT fragments produced by an energy-degraded $^{238}\textrm{U}$ beam on a $^{209}\textrm{Bi}$ target at the FRS Ion Catcher

**DOI:** 10.1140/epja/s10050-026-01914-4

**Published:** 2026-07-13

**Authors:** A. Shraier, P. Constantin, T. Dickel, I. Mardor, A. Mollaebrahimi, D. Amanbayev, S. Ayet San Andrés, J. Bergmann, Z. Ge, E. Haettner, D. Kar, G. Kripko-Koncz, D. Kumar, K. Mahajan, D. J. Morrissey, M. Narang, W. R. Plaß, C. Scheidenberger, M. Simonov, N. Tortorelli, J. Yu, J. Ahokas, B. Amorim, S. Bagchi, M. Bajzek, D. L. Balabanski, K. Botsiou, V. Charviakova, T. Eronen, S. Glöckner, C. Hornung, N. Hubbard, A. Jaries, N. Kalantar-Nayestanaki, A. Kankainen, A. Karmakar, R. Lozeva, J. L. Rodríguez-Sánchez, I. Mitsiou, M. P. Reiter, E. Piasetzky, V. Piau, Zs. Podolyák, R. Prajapat, D. Prajapati, S. Purushothaman, M. Reponen, E. Rocco, J. Ruotsalainen, D. Kostyleva, S. K. Singh, A. Spătaru, A. State, I. Stefănescu, Y. K. Tanaka, L. Trache, L. Welde, J. Zhao

**Affiliations:** 1 Tel Aviv University, Tel Aviv, Israel; 2 Extreme Light Infrastructure-Nuclear Physics, IFIN-HH, Magurele, Romania; 3 II. Physikalisches Institut, Justus-Liebig-Universität, Giessen, Germany; 4 GSI Helmholtzzentrum für Schwerionenforschung GmbH, Darmstadt, Germany; 5 Soreq Nuclear Research Center, Yavne, Israel; 6 The Hebrew University of Jerusalem, Jerusalem, Israel; 7 Helmholtz Research Academy Hesse for FAIR (HFHF), GSI Helmholtz Center for Heavy Ion Research, Giessen, Germany; 8 Department of Physics, IIT-ISM Dhanbad, Jharkhand, India; 9 Department of Chemistry, Michigan State University, East Lansing, USA; 10 Nuclear Energy Group, ESRIG, University of Gröningen, Gröningen, The Netherlands; 11 Ludwig Maximilian University of Munich, Munich, Germany; 12 Lisbon University, Lisbon, Portugal; 13 Instituto de Fisica Corpuscular, University of Valencia, Valencia, Spain; 14 National Centre for Nuclear Research, Warsaw, Poland; 15 University of Jyväskylä, Jyväskylä, Finland; 16 IJCLab, Orsay, France; 17 CITENI, Industrial Campus of Ferrol, University of Coruña, Ferrol, Spain; 18 School of Physics and Astronomy, University of Edinburgh, Edinburgh, UK; 19 Department of Physics, University Of Surrey, Guildford, UK; 20 Astronomy and Physics Department, Saint Mary’s University, Halifax, Canada; 21 Department of Nuclear Physics, “Horia Hulubei” National Institute for Physics and Nuclear Engineering, Magurele, Romania; 22 High-Energy Nuclear Physics Laboratory, RIKEN, Hirosawa 2-1, Wako, Japan; 23 Helsinki Institute of Physics, Helsinki, Finland; 24 DNAP, Tata Institute of Fundamental Research (TIFR), Mumbai, India

## Abstract

An experiment on multi-nucleon transfer (MNT) reactions was performed at the FRS Ion Catcher at GSI-FAIR, where an energy-degraded ^238^U beam reacted with a ^209^Bi target inside the Cryogenic Stopping Cell (CSC). Target-like fragments (TLF) produced in the CSC were extracted and identified using a Multiple-Reflection Time-of-Flight Mass Spectrometer (MR-TOF-MS). The yields of eight mass-identified A = 210 and A = 211 nuclei were analyzed as a function of degrader thickness, which determines the incoming ^238^U beam energy, by comparing their dependence on degrader thickness with that of elastic-scattering products. The results indicated that they are produced in MNT reactions. This experiment is the first in which a broad range of MNT fragments was observed in an ion catcher following beam energy degradation from the relativistic realm to the MNT range, just above the Coulomb barrier. It sets the stage for further MNT experiments that are planned at the FRS Ion Catcher with different targets and unstable secondary ion beams.

## Introduction

One important area of development at radioactive ion beam (RIB) facilities is the search for a production mechanism for exotic, heavy (mass number *A* = 170–260) neutron-rich (n-rich) isotopes [1]. Fission is the preferred production reaction of the medium-heavy n-rich (*A* = 70–170) region [2, 3]. Fragmentation provides significant contributions for heavier n-rich nuclei with $A < 230$ [4]. However, the production cross sections for heavier n-rich isotopes fall off steeply when moving towards neutron-rich nuclei. Multi-nucleon transfer (MNT) reactions are a promising mechanism for the generation of neutron-rich RIBs in the *A* = 170–260 region. An experimental comparison a decade ago demonstrated that MNT isotope production cross sections in this region are higher by several orders of magnitude than those of fragmentation [5].

MNT reactions occur in deep-inelastic binary reactions at energies close to the Coulomb barrier, where projectile and target nuclei come into contact and form a transient, molecule-like dinuclear system (DNS) [6]. Within this configuration, the nuclei remain partially distinct while exchanging nucleons, energy, and angular momentum over a short interaction time on the order of $10^{-21}$–$10^{-20}$ s. The reaction dynamics is governed primarily by the relative motion of the interacting nuclei and the transfer of mass and charge between them, along with additional degrees of freedom such as nuclear deformation and orientation. These determine whether the system evolves toward the formation of a compound nucleus (CN) or decays before full equilibration, producing two fragments after substantial nucleon exchange. During this interaction, multiple protons and neutrons can be transferred between the reaction partners, leading to projectile-like (PLF) and target-like (TLF) fragments with masses significantly different from the entrance channel. The primary fragments are typically produced in excited states and subsequently de-excite via particle emission and $\gamma$ decay, resulting in the final nuclei observed [7].

Realizing this cross-section advantage in practice requires addressing several experimental challenges that remain for RIB facilities employing MNT reactions. The main issues are low extraction efficiency due to smaller acceptance caused by the MNT-specific wide emission angle, restricted isotope production when laser ionization is used to extract the reaction products, and gas cell extraction based on gas flow, forcing a compromise between fast extraction time (small gas volume) and high extraction efficiency (large gas volume). The impact of these issues can be seen in existing MNT-driven RIB facilities, such as KISS at RIKEN [8, 9] and IGISOL at Jyväskylä [10]. In the present work these challenges are addressed by performing MNT reactions with a heavy-ion beam that is initially produced and transported at relativistic energies and subsequently slowed down to the MNT regime just above the Coulomb barrier.

**Fig. 1:**
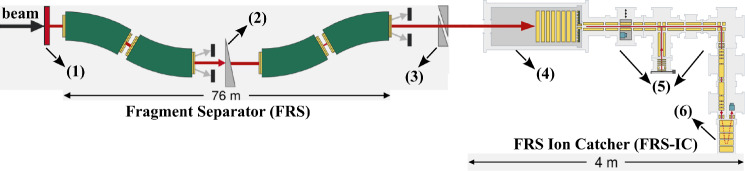
The main components of the experimental setup: (1) beam stripping foil; (2) monochromatic beam degrader; (3) variable beam degrader; (4) CSC with INCREASE setup [16]; (5) RFQ beamline; (6) MR-TOF-MS

We report progress on a research program on MNT-driven secondary beams initiated with the FRS Ion Catcher (FRS-IC, see Fig. 1) at GSI [11], that will be continued with the Super-FRS Ion Catcher at FAIR [12] to address the shortcomings mentioned above [13–15]. A special configuration of the cryogenic stopping cell (CSC) in the FRS-IC has been developed for MNT reactions [16], and early measurements demonstrated its potential [17, 18]. The ultimate goal of the program, utilizing the next-generation CSC at the Super-FRS [12, 19], is to use n-rich secondary beams to induce MNT reactions and generate highly exotic tertiary beams. We note that the KISS facility is engaged in a staged upgrade that will implement similar technologies in the next few years [20], and that another similar setup, the *N* = 126 Factory, is being developed at Argonne National Laboratory (USA) [21].

Along with the main objective to demonstrate the feasibility of using the MNT reactions inside the gas cell to produce RIBs at the FRS-IC, the experiment also addressed several important challenges. First, the mitigation of the space-charge effects inside the gas cell, which disrupts ion motion. A novel beam collection method has shown a large improvement in ion extraction efficiency and transport time of the reaction products inside the gas cell. The INCREASE (IN-Cell REAction SystEm) special configuration of the CSC was implemented [16], as previously described in [15]. This configuration is equipped with a primary space-charge containment system formed by a beam dump and a mini-cage placed at different electric potentials.

Here, we present results from the analysis of the first exploratory experiment of the MNT program at the FRS-IC. The identification and counting processes for the product ions in the Multiple-Reflection Time-of-Flight Mass Spectrometer (MR-TOF-MS) [22] are described. In addition, we investigated the evolution of the number of counts as a function of the thickness of the degrader placed in front of the MNT production target, and thus the reaction energy.

## Experiment

The experiment used a $^{238}$U beam delivered by the SIS18 accelerator at 500 MeV/u and with an intensity around 10$^8$ ions/spill with a typical beam-on time of 10 s and a beam-off time of 4 s. This mimics the conditions expected in future secondary beam experiments. The FRS was used in transmission mode, with a minimum amount of matter. The main components seen by the $^{238}$U beam, shown schematically in Fig. 1, are a 90 mg/cm$^2$ Cu stripping foil, a 737 mg/cm$^2$ monochromatic Al degrader, an air section at the mid-focal and final focal plane (FFP) plane, and a variable Al degrader with a thickness around 1700 mg/cm$^2$ at the FFP. The beam energy on the MNT target was tuned by adjusting the thickness of the variable degrader at the FFP. After entering the gas cell, the beam traversed a 540 mg/cm$^2$ Al plate before hitting the $^{209}$Bi MNT production target, which has a thickness of 49 mg/cm$^2$ with a polyester back holder upstream of it with 17.3 mg/cm$^2$. This final beam slowdown was done close to the target to minimize angular straggling.

The challenge is to slow down the relativistic beam at the entrance of the FRS to a narrow energy range. This range lies a few MeV/u above the Coulomb barrier, where MNT reactions produce the maximum number of transferred nucleons [15]. This capability is also required for future experiments with secondary beams impinging on the MNT target, as these beams will be produced at similar high energies and thus need to be slowed down as well. At the same time, the incoming beam must be focused on the $\simeq 1$ cm (diameter) INCREASE MNT production target, over a distance of about 530 cm from the last FRS quadrupole. For comparison, experiments with the standard CSC configuration only require focusing the beam on 20$\times$10 cm$^2$ windows over a similar distance.

Dedicated tests were performed to demonstrate the required beam energy range and transverse profile on the MNT target. These tests included beam spot identification directly in front of the gas cell using a fluorescence screen, evaluation of the ionization current on the INCREASE beam dump, and optimization of the beam range with stopped beam ions.

The reaction products and elastically scattered ions from the MNT production target are stopped in the gas cell filled with He at 3 mg/cm$^2$ (corresponding to a pressure of 130 mbar and a temperature of 102 K). They are then transported by DC fields to the RF Carpet, where they are guided to a nozzle for extraction by gas flow [23]. A Radio-Frequency Quadrupole (RFQ) beamline is used to apply a coarse mass filter and transport the filtered ions to the MR-TOF-MS [24, 25]. Finally, the MR-TOF-MS [26] is used to identify by mass and to count the ions that reach this stage. In the experiment presented here, this configuration was shown to work at beam intensities of 10$^6$ ions/s, which are expected for the secondary beams available for MNT experiments at the Super-FRS [27].

For each event, the corresponding Time-Of-Flight (TOF) was recorded using a MagneTOF [28] detector and a FAST ComTec TDC (MCS6A) [29]. The spectra were obtained and processed using the TOFControl software [30], which included Time-Resolved Calibration to correct for temporal drifts [31].

## Results

The analysis is based on seven consecutive measurements performed under practically identical FRS-IC settings, the only parameter that varied between the measurements being the thickness of the variable degrader at the FFP.

To identify potential MNT fragments, we measured the mass range of TLF with $$A = 205{-}213$$. The mass numbers $$A = 208 $$ and $$212$$ were dominated by decay products from the $$^{228}\textrm{Th}$$ $$\alpha $$-recoil source located within the CSC for calibration purpose and $$A = 209$$ by the elastically-scattered target nuclei ($$^{209}$$Bi). The long tails of these relatively abundant peaks prevented confident identification and counting of MNT products at these mass numbers.

The mass numbers $A = 205$ and $213$ lie at the edges of the transmitted mass range, where the spectrum is distorted and reliable identification is not possible. The mass numbers $A = 206$ and $207$ have low count rates even at the optimal beam energy. These mass numbers were therefore excluded from further consideration, and the remaining analysis focused on the MNT products identified at $A = 210$ and $211$.

**Fig. 2:**
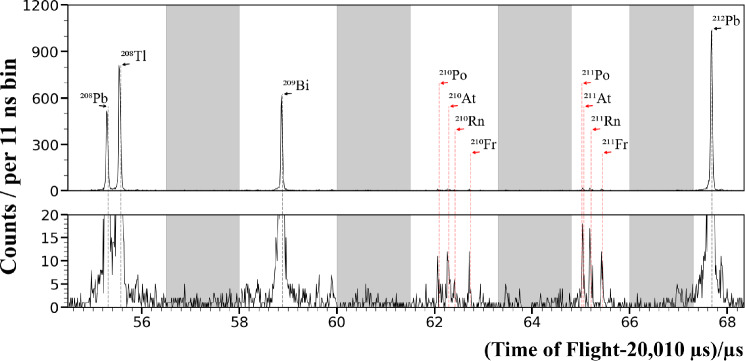
Time-of-flight spectrum accumulated over several degrader settings during a total measurement time of 4 h. The most abundant peaks originate from the elastic channel (^209^Bi) and from daughter nuclei of the ^228^Th $\alpha$-recoil source located inside the CSC. Upper panel: Full counts scale, highlighting the dominant peaks. Lower panel: Low count scale, revealing low-statistics areas where MNT fragments, indicated by red dashed lines, are expected based on their literature mass values [32]. Shaded areas correspond to background estimation regions (see text for details). The horizontal axis is labeled **Time of Flight – 20,010 (µs)** to reflect that the plot describes the full flight time of the ions in the MR-TOF-MS. Note that ions of each mass number shown in the figure traveled a different number of turns in the MR-TOF-MS, from 448 for $A=208$ to 444 for $A=212$

The time-of-flight spectrum shown in Fig. 2, compiled from measurements with several degrader thicknesses, covers this remaining mass range. Isotopes of Po, At, Rn, and Fr were identified. The successful observation of Fr nuclides, corresponding to 4-proton transfer and 2–3-neutron loss from the $^{209}\textrm{Bi}$ target, demonstrates the capability to transfer many nucleons. Based on this, $A = 210, 211$ nuclides of Bi, Pb, and Tl are expected. However, a high level of contamination was present in the stopping gas in the CSC throughout the measurement, due to an air leak. These highly reactive nuclides underwent chemical reactions in the CSC, which reduced their quantity to below the detection and identification limit.

**Fig. 3:**
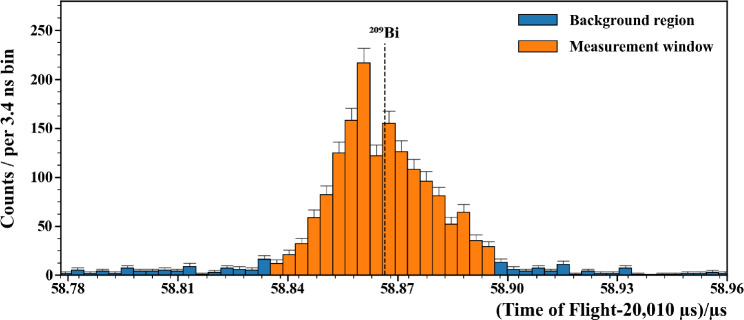
Time-of-flight spectrum around $^{209}$Bi. Events within the predefined $60\,\text {ns}$ integration range are shown in orange and labeled “Measurement window,” while those outside this range are shown in blue and labeled “Background region”. The vertical dashed line marks the position of $^{209}$Bi based on its literature mass value [32]

In order to prove that ions at $A = 210$ and $211$ shown in Fig. 2 are produced via the MNT reaction, the number of counts was analyzed as a function of the FFP degrader thickness, which defines the incoming beam energy. Based on MOCADI simulations [33], the degrader thickness scan corresponds to beam energies on the target, ranging from a few up to about $25\,\mathrm {MeV/u}$. The Coulomb barrier energy for the $^{238}\textrm{U}+{}^{209}\textrm{Bi}$ interaction is approximately $6.8\,\mathrm {MeV/u}$ in the laboratory frame [34–36], setting the lower energy threshold below which MNT reactions are suppressed. The dominant peaks shown in Fig. 2 were used to define the width of the region of interest. The full width at half maximum (FWHM) of the dominant peaks was determined to be $30\,\text {ns}$ by fitting a Gaussian function to the data. See Fig. 3 for a detailed view of the peak shape. The integration range for each species is $\pm 30\,\text {ns}$ around the literature mass.

**Fig. 4:**
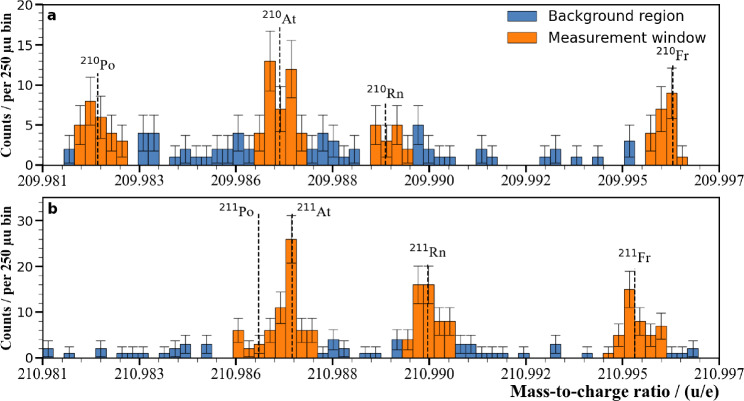
Mass spectrum obtained from the same measurements as in Fig. 2. **a** Corresponds to mass number $A=210$ and **b** to $A=211$. As in Fig. 2, $A=210$ and $A=211$ ions traveled 446 and 445 turns in the MR-TOF-MS, respectively

The measurement was set for 449 isochronous turns (it), corresponding to the center of the mass analyzer at $A = 207$. Since the revolution period scales with the square root of the mass-to-charge ratio, the turn numbers for the neighboring isobars are scaled by the square root of their mass ratios, corresponding to 446 turns for $A = 210$ and 445 turns for $A = 211$ (see the captions of Figs. 2 and 4). The flight time of ions through the MR-TOF-MS for a given number of turns can be expressed as:

1 $$\begin{aligned} t_{\text {total}} = t_{\text {tfs}} + N_{\text {it}} \cdot t_{\text {it}} + t_{0}, \end{aligned}$$

where $t_{\text {total}}$ is the total TOF, $N_{\text {it}}$ is the number of isochronous turns completed by the species of interest in the analyzer, $t_{0} = 0.355~\upmu \text {s}$ is an electronic signal delay, and $t_{\text {tfs}} = 42.43~\upmu \text {s}$ is the TOF between the opening of the exit mirror and the ion detection in the MagneTOF, measured during a zero-turn calibration. The term $t_{\text {it}}$ is the TOF per turn in the analyzer, calibrated using $^{133}$Cs ions, yielding $t_{\text {it}} = 35.7~\upmu \text {s}$.

Using the relation $m/\Delta m = \textrm{TOF}/(2\cdot \Delta \textrm{TOF})$ [31], where *m* is the ion mass, $\Delta m$ is the smallest resolvable mass difference, and $\Delta \textrm{TOF}$ is the corresponding peak width (FWHM), a mass resolving power (MRP) of $\approx 330{,}000$ was achieved with the measured $\Delta \textrm{TOF}$ at FWHM of $0.03~\upmu \textrm{s}$, providing an optimal mode of operation for a wide mass range measurement and robust isotope identification. The mass spectra zoomed for the MNT candidates are shown in Fig. 4. Note, the peaks of $^{211}\textrm{Po}$ and $^{211}\textrm{At}$ have overlapping regions of interest. The ions of interest were detected as singly charged species. Molecular ions with the same nominal mass-to-charge ratio constitute the background, which is estimated and subtracted as described below.

An investigation of the background regions outside the regions of interest revealed that the background increased as the degrader thickness decreased. At the thickest degrader settings, a few elastically scattered $^{238}\textrm{U}$ ions are still detected. Besides elastically scattered targets and projectiles, lighter fragments produced during the slowing-down process can have ranges too large to be stopped in the beam dump; they generate ionization of the stopping gas in the CSC. The resulting ionization leads to the formation of a variety of unknown molecular ion species. These molecular ions appear in the mass spectrum clustered around integer mass numbers, coinciding with the positions where our ions of interest are expected. The events in the shaded regions in Fig. 2 are also due to these molecular ions.

The molecular ion background in the CSC has two contributions: a baseline component present throughout the spectrum, and a beam-induced component that increases with the beam energy deposited in the CSC. The baseline component arises from contamination due to residual gas impurities and an air leak in the CSC during the measurements. The beam-induced ionization elevates the molecular ion background broadly across the spectrum; however, since molecular ion formation produces species whose combined mass sums to near-integer values, this enhancement is most pronounced at integer mass positions, precisely where our ions of interest appear. We assumed that their amount is proportional to the number of elastically scattered ions in the spectrum.

To estimate the baseline component, we sampled the inter-mass regions of the spectrum, indicated by the shaded areas in Fig. 2. These regions fall between integer mass positions where no nuclides are expected. For each shaded region, the number of observed counts $N_{\text {bg}}$ was divided by the width of the region $\Delta t$ and scaled to the width of the integration window used for the ions of interest. This yields a per-region estimate of the baseline component expected under one integration window:

2 $$\begin{aligned} B = \left( \frac{N_{\text {bg}}}{\Delta t}\right) 2\,\langle \text {FWHM}\rangle \end{aligned}$$

where $\langle \text {FWHM}\rangle$ is the mean peak width determined from the dominant peaks in the same measurement. The estimates of five sampled regions were averaged to obtain a single measurement-specific baseline component $\langle B \rangle _i$, where the index i denotes the measurement corresponding to a specific degrader thickness. Its uncertainty was obtained by propagating the Poisson statistical uncertainties of the individual region estimates.

**Fig. 5:**
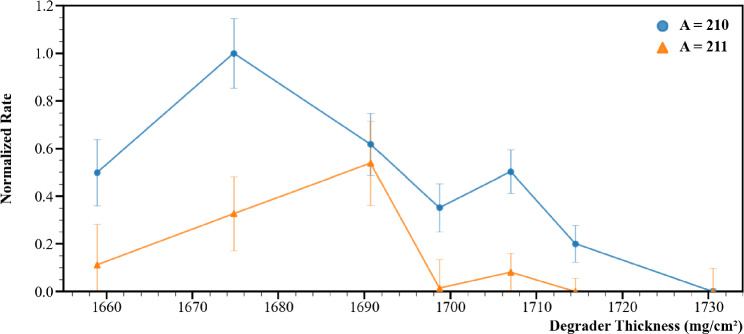
Normalized rates of nuclides with mass numbers $A = 210$ (blue circles) and $A = 211$ (orange triangles) as a function of degrader thickness. A common normalization is applied using the maximum rate observed in the $A = 210$ distribution, allowing a direct comparison of the relative yields. Each data point represents the summed contributions of the isotopes shown in Fig. 4. Connecting lines between points are included for visual guidance and do not represent interpolated values between data points

The baseline component $\langle B \rangle _i$ captures the molecular ion contribution sampled between mass positions, but does not account for the beam-induced enhancement at integer mass positions. To correct for this, a mass-position-specific correction factor $\alpha _j$ is derived from the reference measurement, taken at the thickest degrader setting ($1730.5\,\mathrm {mg/cm^2}$). In this setting, the beam barely begins to exit the target and, therefore, the extraction of MNT TLFs is expected to be negligible. Any counts observed at nuclide $j$’s mass position in the reference measurement therefore originate purely from background, and the correction factor is defined as:

3 $$\begin{aligned} \alpha _j = \frac{N_{j,\textrm{ref}}}{\langle B \rangle _{\textrm{ref}}} \end{aligned}$$

where $N_{j,\textrm{ref}}$ is the number of counts at nuclide $j$’s position in the reference measurement, and $\langle B \rangle _{\textrm{ref}}$ is the baseline component of the reference measurement. The rescaled background for nuclide $j$ in measurement $i$ is then:

4 $$\begin{aligned} \langle B \rangle _{\textrm{rs},j,i} = \alpha _j \, \langle B \rangle _{i} \end{aligned}$$

This correction was applied to all identified nuclides using the same procedure. For the $A=211$ species, counts were observed at their mass positions in the reference measurement, yielding well-defined values of $\alpha _j > 1$, indicating a measurable beam-induced enhancement above the baseline. For the $A=210$ species, no counts were observed at their positions in the reference measurement, consistent with no significant beam-induced enhancement at those mass positions; accordingly, $\alpha _j = 1$ was adopted and the baseline component $\langle B \rangle _i$ was used directly.

For each nuclide $j$ and measurement $i$, the rate $R_{j,i}$ is determined by subtracting the rescaled background from the observed counts and normalizing by the acquisition time $T_i$ and beam intensity $I$:

5 $$\begin{aligned} R_{j,i} = \frac{N_{j,i} - w_j \, \langle B \rangle _{\textrm{rs},j,i}}{T_i \, I} \end{aligned}$$

Where $w_j$ is a window multiplicity factor accounting for cases where the integration windows of adjacent nuclides overlap. For all species $w_j = 1$, except for $^{211}$Po and $^{211}$At, whose $60$ ns integration windows are centered $40$ ns apart and therefore partially overlap. These two windows are treated as a single combined window of $100$ ns, giving $w_j \approx 1.67$, and their counts are taken as a single joint contribution $^{211}$Po + $^{211}$At. The beam intensity $I$ was measured by the SEcondary Electron TRAnsmission Monitor (SEETRAM) in the FRS [37]. The uncertainty in $R_{j,i}$ was estimated by propagating the statistical uncertainties of $N_{j,i}$ and $B_{j,i}$, while the uncertainties in $T_i$ and $I$ were neglected.

The total MNT rate is obtained by summing $R_{j,i}$ over the identified species shown in Fig. 4, with $^{211}$Po and $^{211}$At entering as a single combined contribution: $^{210}$Po, $^{210}$At, $^{210}$Rn, $^{210}$Fr, ($^{211}$Po + $^{211}$At), $^{211}$Rn, and $^{211}$Fr.

**Fig. 6:**
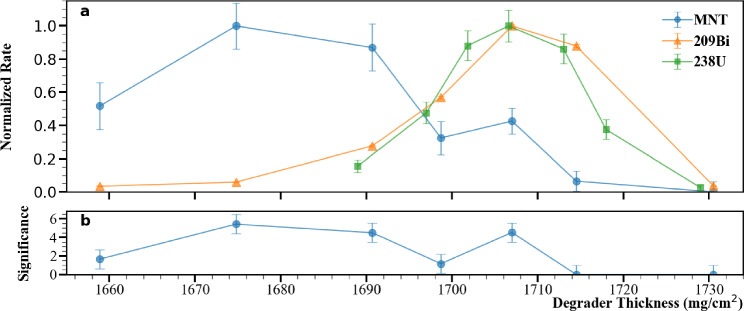
**a** Background-subtracted rates of $^{238}\textrm{U}$ (green squares), $^{209}\textrm{Bi}$ (orange triangles), and the MNT candidates (blue circles), each normalized to their respective maximum values. The MNT candidates represent the summed yields of the mass numbers shown in Fig. 5. **b** The statistical significance of the MNT counts, calculated using Eq.(6). Connecting lines between points in all panels are included for visual guidance

Since MNT production peaks at a few MeV/u above the Coulomb barrier [38], Fig. 5 serves to demonstrate this behavior by showing the rates of $A = 210$ and $A = 211$ as a function of degrader thickness. Both distributions exhibit a broad maximum in a similar thickness region, highlighting their clear dependence on the beam energy. With increasing degrader thickness, incident beam energy is reduced and eventually falls below the Coulomb barrier, leading to suppression of the MNT channel [18]. The fact that both plots follow the same trend provides strong evidence that these groups are produced by the same mechanism. The individual isotopes within each mass group follow the same trend as their summed distribution, with minor differences in the degrader thickness at which their rates peak.

To ensure that the trend observed in Fig. 5 is not an artifact of our experimental system and is unique to MNT processes, we investigated the rate of the primary $^{238}$U beam and the elastically-scattered $^{209}$Bi, shown in Fig. 6 alongside the MNT yields. The MNT and $^{209}$Bi normalized rates were extracted from the same experimental measurements. In contrast, the $^{238}$U rate was obtained from separate measurements of the PLF spectrum, using slightly different degrader thicknesses. In these measurements, the average FWHM of the integration window was determined from six natural Xenon isotope peaks ($^{128,129,130,131,132,134}$Xe) present in the spectrum, following the same procedure described above for the TLF case [39]. These xenon peaks served only to define the integration window and are not reaction products; their measured FWHM of 14–16 ns set the integration range for the $^{238}$U analysis. The TLF and the PLF measurements differed in beam intensity, by nearly two orders of magnitude, and in beam time structure (beam-on and beam-off periods). Since the rates in Fig. 6 are normalized to their respective maxima, these differences do not affect the comparison of their dependence on degrader thickness.

Stopping models estimate a minimum beam energy to penetrate the target around 9 MeV/u (specifically, SRIM [40] gives 8.4 MeV/u and ATIMA [41] gives 9.6 MeV/u), which corresponds to the degrader thickness around 1730 mg/cm$^2$ in Fig. 6, where the $^{238}\textrm{U}$ beam ions and $^{209}\textrm{Bi}$ elastically scattered ions start to escape from the target into the gas. The dependence of their stopping rates on the degrader thickness reflects the wide angular distribution of the beam and target ions stopped inside the ion catcher, driven by multiple elastic scattering inside the target and the setup angular acceptance [15]. Finally, the higher beam energies observed for the MNT rates are due to the need to additionally overcome the Coulomb barrier and compensate for the reaction energy and final state evaporation.

The main background source for the TLFs identified in Fig. 4 could come from beam fragmentation inside the variable degrader. LISE++ simulations [42] show that these isotopes would arrive at the $^{209}\textrm{Bi}$ target with a mean energy higher than the $^{238}\textrm{U}$ beam by 20-25 MeV/u. Hence, their stopping inside the ion catcher requires a thicker degrader, corresponding to these higher energies, and would appear in Fig. 6 to the right of the $^{238}\textrm{U}$ peak. Another argument against this possible source comes from the fragmentation cross sections. Estimates with the EPAX 3 model [4] give a strong isotopic decrease Fr:Rn:At:Po = 9.41:3.72:0.96:0.17 mb. Such a rate variation by a factor more than 50 is not supported by the rates in Fig. 4, which are approximately constant. The only element-dependent efficiency in our setup is the chemical efficiency, but it is not expected to vary so strongly over four charge units.

The statistical significance of the measured counts relative to the background was evaluated using the $Z_{\textrm{Bi}}$ method, following [43]. In this approach, the problem is formulated as a test of the background-only hypothesis in the presence of background uncertainty. By conditioning on the total number of counts, the Poisson counting problem is reduced to a binomial distribution, from which the probability $P_{\textrm{Bi}}$ is calculated for the background to produce a fluctuation equal to or larger than the measured counts. This probability is then expressed as a Z-value:

6 $$\begin{aligned} Z_{\textrm{Bi}} = \sqrt{2}\,\textrm{erfc}^{-1}(2P_{\textrm{Bi}}) \end{aligned}$$

The significance was calculated using the total counts obtained by summing all nuclides that contribute to the MNT yield, as shown in Fig. 4. The corresponding background and its uncertainty were obtained by summing the individual background contributions of the same nuclides. The uncertainty in $Z_{\textrm{Bi}}$ was estimated via standard error propagation.

Figure 6b presents the resulting significance as a function of degrader thickness. In the thickest degrader setting, the observed yields are consistent with background, resulting in a significance compatible with zero. As the degrader thickness is varied in the region where MNT production is observed, the significance increases, reaching values of $Z_{\textrm{Bi}} \approx 4.5$, 4.4 and up to 5.4, indicating a clear excess of counts above the background. For thinner degrader settings, the significance decreases again, consistent with the reduction of the MNT yield outside the optimal energy range. This behavior follows the expected threshold and energy dependence of the MNT process and supports the identification of the observed signal as originating from MNT reactions.

## Summary and conclusions

A $^{238}\textrm{U}$ beam was bombarded on a $^{209}\textrm{Bi}$ target inside the CSC at the FRS Ion Catcher at GSI, with beam energies just above the Coulomb barrier that enable MNT reactions using the INCREASE device [16]. The resulting fragments were extracted and identified using an MR-TOF-MS. Results from the analysis of data focused on TLFs at the mass region $A = 210$ and $211$ are presented. The data analysis procedure included time-resolved calibration, mass calibration, normalization to beam intensities, mass identification, and background characterization. The data analyzed here included seven measurements with similar conditions except for variation of the FFP degrader, which altered the incoming beam energy.

The rate of MNT candidates was found to be centered at significantly lower degrader thicknesses (namely, higher beam energy) than those of elastically scattered $^{238}\textrm{U}$ and $^{209}\textrm{Bi}$ ions. This proves that these candidates are indeed products of MNT reactions. The statistical significance of the MNT yields was found to be more than 4 standard deviations above background, ensuring that they are not a result of background statistical fluctuations.

The analysis of the data in this work sets the stage for a detailed analysis of the entire data set from this experiment. The subsequent analysis will include the identification of more MNT fragments. Furthermore, in order to extract MNT production cross sections we will follow an approach similar to that used for isotopic fission yield measurements at the FRS Ion Catcher [44]. Additional MNT experiments at the FRS Ion Catcher include a measurement with a secondary $^{236}\textrm{U}$ beam, which has already been performed and is currently under analysis. A second experiment, using a $^{238}\textrm{U}$ primary beam on a $^{238}\textrm{U}$ target, is scheduled to take place in the near future.

## Data Availability

Data will be made available on reasonable request. [Authors’ comment: The datasets generated during and/or analysed during the current study are available from the corresponding author on reasonable request.]

## Code Availability Statement

Code/software cannot be made available for reasons disclosed in the code availability statement. [Authors’ comment: The code/software generated during and/or analysed during the current study is not publicly available due to it being built on an internal, collaboration-specific analysis framework but is available from the corresponding author on reasonable request.]
